# Collaborative Purification of Tert-Butanol and N_2_O over Fe/Co-Zeolite Catalysts

**DOI:** 10.3390/ijerph20064902

**Published:** 2023-03-10

**Authors:** Ruiqi Wu, Ning Liu, Chengna Dai, Ruinian Xu, Ning Wang, Gangqiang Yu, Biaohua Chen

**Affiliations:** Faculty of Environment and Life, Beijing University of Technology, Beijing 100124, China

**Keywords:** N_2_O, tert-butanol, catalytic oxidation, molecular sieve

## Abstract

N_2_O is a greenhouse gas and a candidate oxidant. Volatile organic pollutants (VOCs) have caused great harm to the atmospheric ecological environment. Developing the technique utilizing N_2_O as the oxidant to oxidize VOCs to realize the collaborative purification has significant importance and practical value for N_2_O emission control and VOC abatement. Therefore, the study of N_2_O catalytic oxidation of tert-butanol based on zeolite catalysts was carried out. A series of molecular sieves, including FER, MOR, ZSM-5, Y, and BEA, were selected as the catalyst objects, and the 1.5% wt Fe and Co were, respectively, loaded on the zeolite catalysts via the impregnation method. It was found that the catalytic performance of BEA was the best among the molecular sieves. Comparing the catalytic performance of Fe-BEA under different load gradients (0.25~2%), it was found that 1.5% Fe-BEA possessed the best catalytic activity. A series of characterization methods showed that Fe^3+^ content in 1.5% Fe-BEA was the highest, and more active sites formed to promote the catalytic reaction. The α-O in the reaction eventually oxidized tert-butanol to CO_2_ over the active site. The Co mainly existed in the form of Co^2+^ cations over Co-BEA samples; the 2% Co-BEA possessing higher amounts of Co^2+^ exhibited the highest activity among the prepared Co-BEA samples.

## 1. Introduction

Volatile organic compounds (VOCs) are another kind of major pollutant that harm the environment, in addition to particulate matter, nitrogen oxides, and sulfur dioxide. Most VOCs have toxic effects on human health and the natural environment. After exposure to VOCs, people may experience symptoms such as dizziness, nausea, coughing, and foreign body sensation in the eyes. If humans live in places where VOCs are emitted for a long time, VOCs will damage the central nervous system and liver of the human body [1]. When several toxic VOCs coexist, the harm of their combined action is much greater than that of single VOCs. Under sunlight, VOCs can photochemically react with nitrogen oxides in the atmosphere to generate photochemical smog, resulting in secondary pollution. In recent years, with the development of my country’s industry, the emission of VOC pollutants has increased, which has caused more and more serious ecological problems. Therefore, how to effectively manage VOCs has become the focus of the society.

There are many traditional VOC removal methods; the ones commonly used are the absorption method, the adsorption method, the condensation method, the direct combustion method, the catalytic oxidation method, and the biological method [2].

Catalytic oxidation is a method for oxidizing VOCs into CO_2_, H_2_O, and other harmless compounds, so catalytic oxidation is known as one of the most effective and economical methods. The catalytic oxidation method can completely destroy VOCs, rather than converting them into another phase for processing such as adsorption and condensation [3]. Compared with the direct combustion method, the thermal efficiency of catalytic oxidation is higher, and a lot of combustion heat energy is not wasted. Catalytic oxidation is more suitable for low-concentration VOC industrial exhaust treatment, so it is widely used in terminal VOC pollution control. One of the main challenges in the catalytic degradation of volatile organic compounds is the selection of suitable catalysts from the large number of available catalysts.

In the practical application of catalytic oxidation, the most important thing is to choose a suitable catalyst. In the research of catalytic oxidation of VOCs, the commonly used catalysts are roughly divided into the following three categories: metal oxide catalysts, supported noble metal catalysts, and molecular sieve catalysts. There is also much research on these three types of catalysts, and many achievements have been obtained [4,5].

The zeolite molecular sieve is an important inorganic porous material with an abundant pore structure and large specific surface area. Since the discovery of natural zeolite in 1756, many researchers have continued to explore and have discovered analcite, sodalite, and mordenite. With the development of industry, many artificial zeolite Y-type, L-type, MOR, and other molecular sieves were gradually synthesized, and also widely used in industrial catalysis. There are many types of molecular sieve catalysts. The molecular sieves can be found in the International Molecular Sieve Association. In the process of the practical application of molecular sieves to catalyze VOCs, the metal (usually a transition metal) will be loaded on the molecular sieve for modification to obtain better catalytic activity [6,7,8,9].

In 2015, Ágnes Szegedi et al. [10] prepared cobalt-modified MCM-41 and SBA-15 mesoporous materials by adjusting the pH of the immersion solution, and found that these materials were active for the oxidation of toluene. In 2016, the results of M. Romero-Sáez et al. [11] showed that Fe-ZSM-5 prepared via the impregnation method or solid-phase ion exchange method was active for the oxidation of trichloroethylene (TCE). However, the comprehensive activity of the catalysts synthesized by the two methods is not as good as that of the ion exchange method. In 2017, the activity evaluation experiment of Zhen Cheng et al. [12] showed that the 10%MnCo(6:1)/MCM-41 (Mn/Co molar ratio was 6:1) catalyst exhibited the best catalytic activity, and chlorobenzene (1000 ppm) could be completely decomposed at 270 °C. In 2020, Wei Zhao et al. [13] modified the ZSM-5 molecular sieve via the impregnation method of Co, and lowered the temperature of n-butane oxidation on traditional ZSM-5 from 574 °C to 374 °C.

The catalytic oxidation of VOCs also requires the selection of appropriate oxidants to oxidize VOCs into harmless gases or substances. Common oxidants are O_2_, H_2_O, H_2_O_2_, HNO_3_, and N_2_O. Among them, N_2_O is used as an oxidant and is also a common industrial exhaust gas, which has attracted the attention of researchers [14,15]. N_2_O is the third largest greenhouse gas, and the potential greenhouse effect of N_2_O is about 310 times that of CO_2_ and 21 times that of CH_4_. It has a lifespan of about 150 years in the atmosphere. Over the years, scientists have found that N_2_O is a mild oxidant in research. It has excellent performance in the preparation of phenol via the direct oxidation of benzene, and the yield of phenol is very high. In adipic acid and nitric acid plants, the tail gas N_2_O can be directly utilized as a resource to produce phenol as an industrial raw material [16]. In 1988, Suzuki [17] and his colleagues began to study the preparation of phenol via N_2_O oxidation of benzene over a ZSM-5 molecular sieve catalyst. In the reaction, it was found that the selectivity of phenol could be increased to 70%. Many subsequent pieces of literature published studies on the one-step oxidation of benzene to phenol using N_2_O as an oxidant on ZSM-5, including studies on the reaction mechanism, active centers, and deactivation of carbon deposition [15,18,19]. Panov et al. [20] found that Fe loading on the ZSM-5 catalyst could significantly improve the activity of N_2_O oxidation of benzene to phenol. The experimental results showed that the conversion rate of benzene is as high as 25~30%, and the selectivity of phenol is close to 100%.

In 2014, R. Navarro et al. [21] found a 22% yield of phenol on 1 wt% Fe dibasic phosphate at 350 °C, in which highly dispersed iron species were present. In 2016, lulu Li [22] synthesized graded Fe-ZSM-5 molecular sieves with microsphere morphology via the partial crystallization method using 3-glycidyloxypropyltrimethoxysilane (GPTMS) as the raw material. This graded Fe-ZSM-5 had a mesoporous-rich microsphere morphology, strong catalytic activity, and good stability in the N_2_O oxidation of benzene to phenol. In 2019, Cui Ouyang [23] used a variety of Fe-ZSM-5 catalysts to study the oxidation of benzene to phenol (BTOP) using N_2_O as the oxidant. The initial selectivity of phenol was as high as 95.9%, and the concentration of Lewis acid sites on the catalyst surface was 0.021 mmol g^−1^.

In addition, compared to other oxidants, N_2_O is present in many industrial exhaust gases. These exhaust gases not only contain N_2_O, but also VOCs such as benzene, toluene, dichloroethane, n-butane, and tert-butanol [14]. If N_2_O can be used as an oxidant to react with these organic compounds, it can not only remove the greenhouse gas N_2_O and convert it into O_2_ and N_2_ [24], but it can also be used to remove the harmful VOCs in the tail gas. It can purify two kinds of harmful substances in coordination, and also conforms to the current green environmental protection concept. The main research content of this paper focused on the oxidation of tert-butanol (C_4_H_10_O) in VOCs with N_2_O as the oxidant. Five molecular sieves FER, MOR, ZSM-5, Y, and BEA were selected and modified via the impregnation method. This simulated industrial caprolactam tail gas proportioning the atmosphere. A small amount of oxygen was added to the reaction system of N_2_O and tert-butanol to control the coking of the molecular sieve [25] and to improve the conversion of N_2_O. The conversion rate and selectivity were analyzed according to the experimental results, and the molecular sieve catalyst with the best catalytic activity was finally selected. The structure–activity relationship was explained through the result of characterization.

## 2. Materials and Methods

### 2.1. Catalyst Preparation

The five kinds of parent H-zeolite catalyst (FER, MOR, ZSM-5, Y, and BEA) were commercial products provided by Nankai Catalyst Factory. The SiO_2_/Al_2_O_3_ ratio of all zeolites was 25. The modified metals Fe and Co were loaded on the zeolite catalysts via the impregnation method with the metallic concentration ranging from 0.25% to 2%. Metallic nitrates (Fe (NO_3_)_3_·9H_2_O and Co(NO_3_)_2_·6H_2_O) were the source of modified metals Fe and Co, which were purchased from Shanghai Aladdin Biochemical Technology Company.

The experimental steps were as follows: H-zeolites were roasted in the muffle furnace at 550 °C for 8h; a certain amount of metallic nitrates (Fe(NO_3_)_3_·9H_2_O or Co(NO_3_)_2_·6H_2_O) and 5 g H-zeolites were added into 200 mL deionized water under stirring at 90 °C for 4 h; thereafter, the mixture was carried in a rotary vacuum evaporator until the liquid was completely evaporated; and the final product was dried at 100 °C for 8 h in the oven and subsequently calcined at 550 °C for 8 h in the muffle furnace. All samples of the obtained catalyst were finally labeled as x% Fe-zeolite or x% Co-zeolite, where x represents the loading of metal.

### 2.2. Catalytic Performance Tests

The catalyst activity was tested in a quartz fixed-bed reactor (id = 7 mm, od = 9 mm, L = 475 mm) loaded with 0.5 g (40~60 mesh). The reaction atmosphere consisted of 0.2 vol% C_4_H_10_O, 7 vol% N_2_O, and 5 vol% O_2_ balanced by He simulating gas composition in industry. C_4_H_10_O is solid state at room temperature of 25 °C; so, another helium stream was used to blow the heated C_4_H_10_O in the water bath, and then it carried C_4_H_10_O into the line with access into the steel pipe wrapped with 100 °C heating belt for the reaction. The reaction conditions were C_4_H_10_O:N_2_O:O_2_:He = 0.2:7:5:87.8. The gas hourly space velocity (GHSV) was controlled at 8000 h^−1^, while the total flow was set to be 66.67 mL/min. Before testing the catalyst activity, the catalyst samples was pretreated with 30 mL/min helium at 550 °C for 80 min. After pre-treatments, the catalytic oxidation of tert-butanol began.

The reaction temperature range of the test was 300~600 °C with increasing steps of 50 °C. The reactants and products were analyzed using a two gas chromatograph (EWAI GC-4000A). One was equipped with two TCD detectors, propark QS and 5A molecular sieve stainless steel filled column. The other was equipped with two FID detectors, TDX-01 and 10% PEG-20M stainless steel filled column.

### 2.3. Catalyst Characterization

The X-ray diffraction (XRD) measurements were performed on a Bruker D8-Advance equipped with Cu Kα X-ray radiation (λ = 1.5406 Å). The angle ranges of the test were 2θ = 5~60° in the patterns. XRD tests were used to identify changes in crystal structure after loading different concentrations of metals on H-BEA catalysts.

The X-ray photoelectron spectra (XPS) measurements were executed using a Thermo Scientific K-Alpha^+^ with a monochromatized Al Kα source (1486.6 eV, 15 KV, and 15 mA). The vacuum of the analysis room was about 5 × 10^−9^ mbar. XPS was mainly used to determine the metal species Fe and Co in modified zeolite catalysts, so as to study the structure and the distribution of surface atomic valence states. In addition, the peaks of XPS required carbon correction using a standard value of C1s which was 284.8 eV.

The surface area, micropore volume, and average pore diameter were determined via a Micromeritics ASAP 2020HD88 physisorption instrument using N_2_ adsorption/desorption at −196 °C. Prior to determining, the catalyst sample was degassed in a vacuum at 300 °C for 6 h. In the nitrogen atmosphere, the instrument carried out the desorption and adsorption experiment, and obtained the adsorption amount of the adsorbed substance at a relative pressure (P/P_0_) of 0.99. The specific surface area was obtained via the BET method. The micropore volume was calculated using the t-plot equation, the micropore distribution was calculated using the HK model, and the mesoporous distribution was calculated using the BJH model.

The model of Shimadzu UV3600 was used to determine the UV−vis diffuse reflectance spectrum. The absorbance of the molecular sieve powder was determined by using the diffuse reflection integral sphere mode to determine the existence and valence state of metal elements.

The temperature-programmed reduction of hydrogen (H_2_−TPR) was recorded using a TP-5080B adsorber from Xianquan. The signal of the equipment was detected using a TCD detector as an adsorption instrument. The 100 mg zeolite catalyst sample was first loaded into a micro reactor. Afterwards, the sample was preheated at 100 °C to baseline stable and purged with 30 mL/min H_2_/Ar (5 vol% H_2_). Then, the device was programmatically heated at a rate of 10 °C/min and kept at 900 °C for 10 min. In the whole experiment, the bridge current was 50 mA.

## 3. Results and Discussion

### 3.1. Characterization

#### 3.1.1. XRD

**Figure 1:**
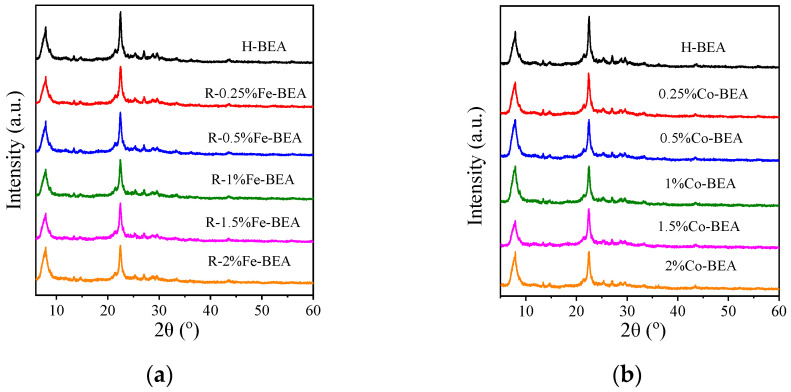
(**a**) XRD patterns of Fe-BEA with different concentrations; (**b**) XRD patterns of Co-BEA with different concentrations.

Figure 1a shows the XRD patterns of Fe-BEA samples with loading capacity of 0.25~2% Fe and H-BEA. The powder XRD patterns of Co-BEA are shown in Figure 1b. Compared with the characteristic peaks of H-BEA, the intensity of the Fe-BEA and Co-BEA peaks decreases slightly at 2θ = 7.8, 22.4, and 25.3°. However, the positions of the characteristic peaks remain unchanged. This indicates that the framework structure of the BEA zeolite does not change after modification by metals Fe or Co. For the XRD pattern of Fe-BEA, there are no typical peaks of some iron oxide species at 2θ = 21.5 and 29.0° [26,27,28,29,30]. For Co-BEA, characteristic peaks of Co_3_O_4_ are unobserved at 19.0, 37.0, and 45.5° [13,26,31]. The oxides of Fe and Co cannot be detected because the metal concentration of the load on the BEA zeolite is too low to reach the detection limit of XRD.

#### 3.1.2. N_2_ Adsorption/Desorption

**Figure 2:**
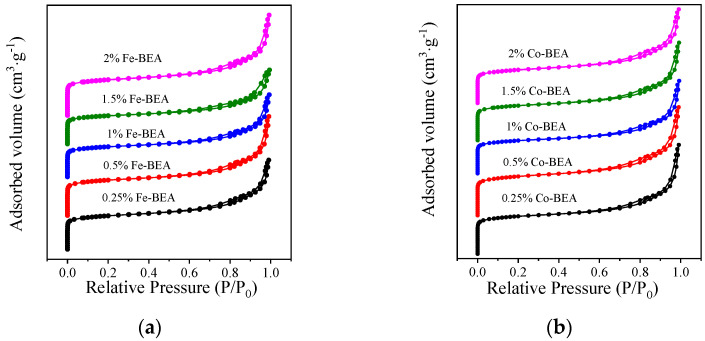
(**a**) N_2_ adsorption–desorption profiles of Fe-BEA with different concentrations; (**b**) N_2_ adsorption–desorption profiles of Co-BEA with different concentrations.

The specific surface area, micropore volume, and average pore diameter of the catalyst samples are listed in Table 1. Analyzing the results in Table 1, it is found that the specific surface area of modified catalysts somewhat decreased compared with H-BEA [26]. The micropore volume also showed a similar trend. The reason for this is that Fe-BEA is prepared via impregnation, and a small amount of iron ions are impregnated in the BEA zeolite. This enables iron ions to enter the zeolite and block the pores of the BEA, as they do in Co-BEA. In addition, the structures of the studied zeolites are all on the nanoscale, and the introduction of cobalt and iron into BEA did not change the overall structure of the zeolite, which can also be confirmed from the XRD analysis above. The structural properties of the catalysts were determined via the N_2_ adsorption method [13], and the N_2_ adsorption and desorption curves are shown in Figure 2. When the relative pressure is higher than 0.68, the N_2_ adsorption and desorption curves on both Fe-BEA and Co-BEA form narrow hysteresis loops, as seen in Figure 2. It can be considered that the curves are both type IV adsorption isotherms, indicating the existence of mesoporous structures in the tested catalyst samples of Fe-BEA and Co-BEA.

#### 3.1.3. XPS

**Figure 3:**
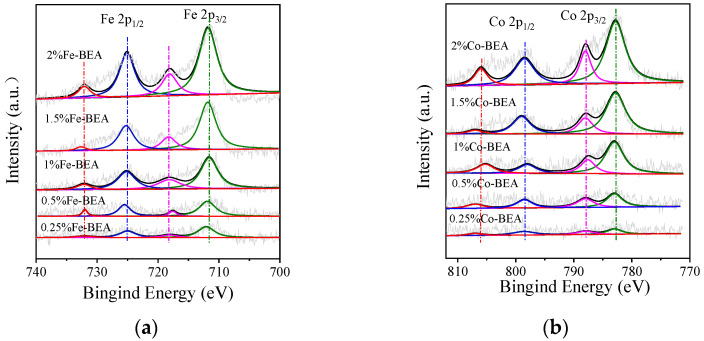
(**a**) XPS spectra of Fe-BEA in Fe 2p orbitals; (**b**) XPS spectra of Co-BEA in Co 2p orbitals.

The XPS spectrum of Fe-BEA with various iron contents in Fe 2p is shown in Figure 3a. The deconvolution peaks of Fe-BEA samples are shown, and it can be seen that the major peaks of Fe 2p_3/2_ and Fe 2p_1/2_ appear at 711.8 eV and 725.2 eV. The satellite peaks of Fe 2p_3/2_ and Fe 2p_1/2_ appear at about 718.1 eV and 732.2 eV. Among them, the peaks at 711.8 eV and 718.1 eV are attributed to the Fe^3+^ species on the surface of the molecular sieve, including Fe^3+^ and Fe_2_O_3_ [32]. The peaks at 725.2 eV and 732.2 eV can be assigned to the electron-binding energy spectrum peaks of Fe 2p_1/2_ of Fe^3+^. It can be seen that the iron element on the surface of Fe-BEA mainly exists in the state of Fe^3+^, mainly including Fe^3+^ and Fe_2_O_3_ [32,33,34]. Figure 3b shows the XPS spectrum of five Co-BEA in Co 2p. For the Co-BEA samples, there are two main peaks in the binding energy of about 782.9 and 798.2 eV, consistent with the Co 2p_3/2_ and Co 2p_1/2_ spin−orbit peaks. According to the literature, it can be shown that 798.2 eV and 807.9 eV belong to Co_3_O_4_ and Co^3+^, while 782.9 eV and 787.6 eV are the peaks of the binding energy of Co^2+^ species. This result indicates that the cobalt element on the surface of Co-BEA with five concentrations mainly exists as Co^2+^ species [33,35]. Meanwhile, it can be seen in Figure 3 that the loadings of metallic Fe and Co have important effects on the surface composition of the Fe-BEA and Co-BEA catalysts.

#### 3.1.4. UV−Vis

**Figure 4:**
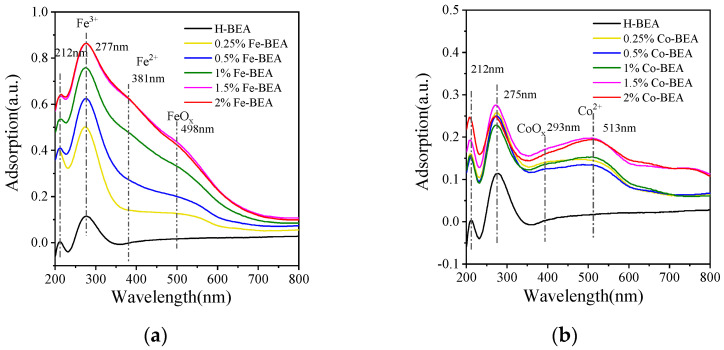
(**a**) UV−vis diffuse reflectance spectra of Fe-BEA with different concentrations; (**b**) UV−vis diffuse reflectance spectra of Co-BEA with different concentrations.

In order to determine the existing forms and valence states of metal elements on the zeolite samples, ultraviolet diffuse reflectance spectroscopy experiments were carried out. Figure 4a shows the experimental data of UV diffuse reflectance spectra of H-BEA: 0.25, 0.5, 1, 1.5, and 2% Fe-BEA. By analyzing the UV absorption peaks of the H-BEA and Fe-BEA samples, some conclusions can be drawn. The UV diffuse reflectance peaks at 212 nm and 277nm should be the characteristic peaks of the BEA zeolite framework. Compared with the peak of H-BEA, the ultraviolet absorption peak of Fe-BEA (0.25% Fe~2% Fe) at 277 nm is more significant. This means that in addition to the framework peak of BEA, there may be other UV diffuse reflection peaks at 277 nm. According to the literature, the peak at 277nm of Fe-BEA is also attributed to Fe^3+^ [30]. In addition, the H-BEA purchased is a commercial molecular sieve, which may retain a small amount of metal ions. Furthermore, it can be found that 1, 1.5, and 2% Fe-BEA have a small peak at 381 nm, which is assigned to Fe^2+^. In the literature, the characteristic UV peak of FeO_x_ with large particles generally appears after 400 nm. Therefore, the UV diffuse reflectance peak at 498 nm is attributed to the UV diffuse reflectance peak of FeO_x_ [30]. Through the comparison of each peak area, it can be found that the iron on the Fe-BEA zeolites mainly exists in the form of Fe^3+^.

The UV diffuse reflectance spectra of Co-BEA are shown in Figure 4b. Consistent with the above, the UV diffuse reflectance peaks at 212 and 275 nm should be the characteristic peaks of UV diffuse reflectance of the BEA zeolite framework. At 293 nm, the Co-modified BEA appears to have a small UV diffuse reflection absorption peak, which is due to the existence of CoO_x_. The area of this peak is very small, indicating that the amount of CoO_x_ is also very small in Co-BEA. The UV diffuse reflection peak at 513 nm is attributed to the UV diffuse reflection peak of Co^2+^ [30]. According to the comparison of the UV absorption peaks of Co-BEA and H-BEA, it can be found that the area of UV absorption peaks at 513 nm changes the most. Therefore, the cobalt mainly exists as Co^2+^ on the Co-BEA zeolite samples.

#### 3.1.5. Temperature−Programmed Reduction by Hydrogen

**Figure 5:**
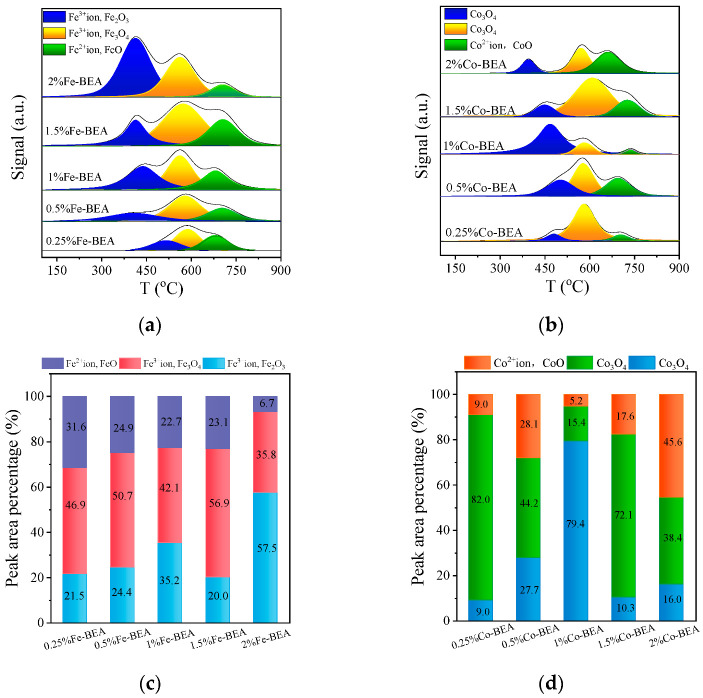
(**a**) H_2_−TPR profiles of Fe-BEA with different concentrations; (**b**) H_2_−TPR profiles of Co-BEA with different concentrations; (**c**) proportion of species corresponding to hydrogen consumption peak of Fe-BEA; (**d**) proportion of species corresponding to hydrogen consumption peak of Co-BEA.

The active metal components of the catalyst were reduced by H_2_ in the H_2_−TPR experiment, in order to characterize the oxidative properties of the catalyst. The H_2_−TPR profiles of Fe-BEA with different concentrations are depicted in Figure 5a. The attribution of iron species is determined by different peak positions. Figure 5c shows the percentage of species content in Fe-BEA. In Figure 5a, there are three reduction peaks in the H_2_−TPR experimental results of Fe-BEA. The position of the peaks changes slightly with the loading iron concentration, but the order of the three reduction peaks does not change. The first two peaks appear at around 450 °C and 600 °C, respectively. The first peak is assigned to the reduction peak of a large number of Fe^3+^ ions and a small amount of Fe_2_O_3_, while the second peak is assigned to the reduction peak of a small amount of Fe^3+^ ions and a large number of Fe_3_O_4_. The third peak appears around 700 °C, which is mainly the reduction peak of Fe^2+^ ions and FeO. It can be clearly seen that Fe^3+^ is the main form of Fe-BEA [26,29]. Additionally, the oxides are a small amount compared to the isolated Fe^3+^ species. This can also be demonstrated with the other characterizations above. As mentioned above, the Fe^3+^ cation as the active center has higher activity than FeO_x_ in the catalytic oxidation of tert-butanol. Therefore, the following discussion focuses on the peak of Fe^3+^. The reduction peak of a large number of Fe^3+^ species is at 600 °C, and that of a small number of Fe^3+^ species is at 450 °C. It is found that the first reduction peak in 2% Fe-BEA contains more oxides, which will deposit on the surface of zeolite and further affect the catalytic performance of zeolite [36,37]. Therefore, 1.5% Fe-BEA contains more Fe^3+^, and its catalytic performance is better, as discussed in Section 3.2.3. As mentioned above, the Fe^3+^ cation as the active center has higher activity than FeO_x_ in the catalytic oxidation of tert-butanol.

Figure 5b displays the H_2_−TPR profiles of Co-BEA with various concentrations. Figure 5d shows the percentage of species content in Co-BEA. The first two peaks are both attributed to the reduction peak of Co_3_O_4_ in Figure 5b. The first peak is around 450 °C, which is the H_2_ reduction peak of Co_3_O_4_ outside the zeolite pores. The other reduction peak of Co_3_O_4_ appears around 600 °C, which is the reduction peak of Co_3_O_4_ inside the pores of the zeolite. The metal species change process is that Co_3_O_4_ is reduced to Co^2+^ at the first two peaks. Co^2+^ species are reported in the literature to be difficult to reduce [26,29,35,38]. Therefore, this usually occurs in the high temperature section. The reduction peak of Co^2+^ and CoO appears around 730 °C in Figure 5b, which is reduced to Co^0^. According to the proportion of Co^2+^ species, it can be concluded that the reduction peak of Co^2+^ species in 2% Co-BEA is the largest compared with the experimental results.

### 3.2. Activity Measurement

#### 3.2.1. Activity Comparison on Different Fe Zeolite Catalysts

**Figure 6:**
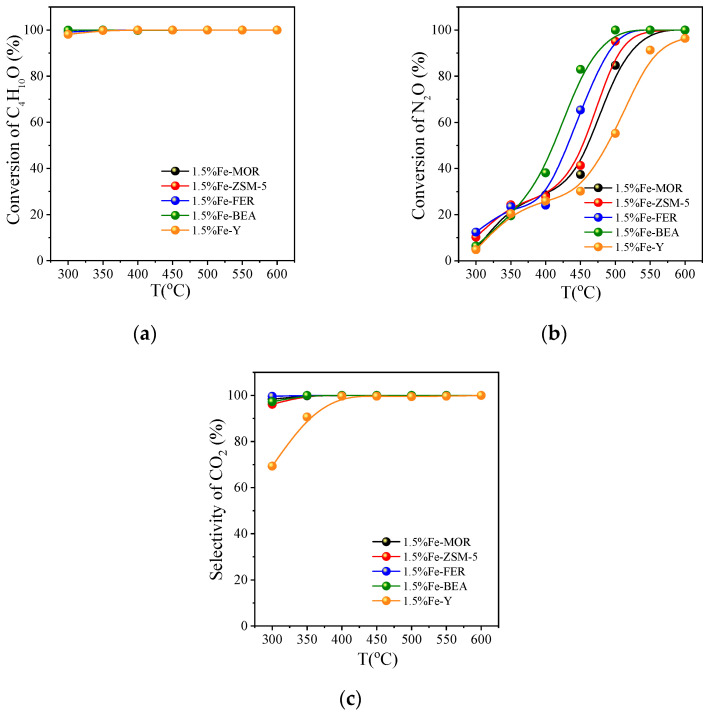
Catalytic oxidation of tert-butanol on zeolite catalysts loaded with 1.5% Fe: (**a**) the conversion of C_4_H_10_O; (**b**) the conversion of N_2_O; (**c**) the selectivity of CO_2_; reaction conditions: GHSV = 8000 h^−1^, C_4_H_10_O:N_2_O:O_2_:He = 0.2:7:5:87.8.

In a series of evaluation experiments, the catalytic activity of zeolites loaded with 1.5% Fe was first evaluated. These five iron-based zeolites include FER, MOR, ZSM-5, Y, and BEA. This was evaluated by the conversion of tert-butanol, the conversion of N_2_O, and the selectivity of CO_2_, as shown in Figure 6a–c. The catalytic activity of iron-based zeolites in the catalytic oxidation of tert-butanol varies greatly depending on the types of zeolites. Figure 6a depicts the change in the conversion of tert-butanol on zeolites with different configurations loaded with 1.5% Fe in the range of 300~600 °C. It is found that the conversion rates of tert-butanol on the five zeolites all reach more than 98% at 300 °C. Moreover, the conversion rate of tert-butanol on different zeolites is not much different. Therefore, it is necessary to compare the other two catalytic activity indicators. Figure 6b shows the conversion of N_2_O on different catalysts, and it is found that the conversion of N_2_O increases with an increase in temperature; at 400~450 °C, it can be clearly seen that the catalytic activity of 1.5% Fe-BEA is better than that of the other four zeolites. The conversion of N_2_O on 1.5% Fe-BEA reaches 83.0% at 450 °C and the conversion of N_2_O reaches 100% at 500 °C.

At 300~350 °C, some of the catalysts in Figure 6c produced CO, a small amount of propylene and isobutylene (Supporting Figure S3), resulting in low CO_2_ selectivity. The reason for the generation of CO and other products is the insufficient reaction in the low-temperature section, so that C_4_H_10_O cannot be completely converted into CO_2_. In summary, the catalytic activity of the catalyst 1.5% Fe-BEA is significantly better than that of the other four molecular sieves. To explore further, we also compared the catalytic activity of the catalysts loaded with 1.5% Co in the following section.

#### 3.2.2. Activity Comparison of Different Co Zeolite Catalysts

**Figure 7:**
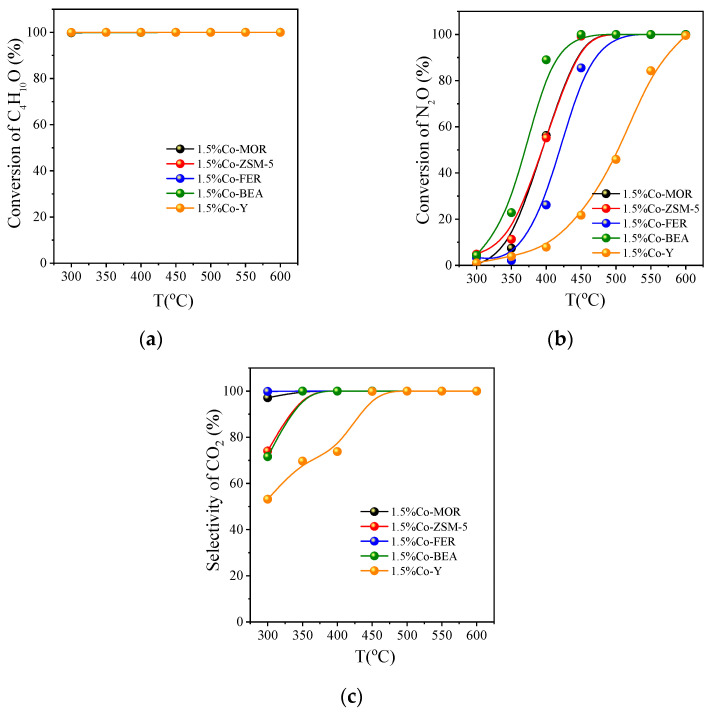
Catalytic oxidation of tert-butanol on molecular sieves loaded with 1.5% Co: (**a**) the conversion of C_4_H_10_O; (**b**) the conversion of N_2_O; (**c**) the selectivity of CO_2_; reaction conditions: GHSV = 8000 h^−1^, C_4_H_10_O:N_2_O:O_2_:He = 0.2:7:5:87.8.

In Figure 7a, the conversion of tert-butanol is not much different to the catalyst loaded with 1.5% Co. However, there is a clear difference in the conversion of N_2_O in Figure 7b. The catalytic activity of N_2_O on 1.5% Co-BEA is the best; by 400 °C, it has reached 89.0%. The order of N_2_O activity on the five molecular sieves is as follows: 1.5% Co-BEA > 1.5% Co-MOR > 1.5% Co-ZSM-5 > 1.5% Co-FER > 1.5% Co-Y. Some CO, a small amount of propylene and isobutylene (Supporting Figure S3) are generated at 300 °C with 1.5% Co-BEA, 1.5% Co-MOR, 1.5% Co-ZSM-5, and 1.5% Co-FER, as seen in Figure 7c. The same substance is also produced in 1.5% CoY at 300~400 °C, which reduce the selectivity of CO_2_. In conclusion, the best catalytic activity for a 1.5% cobalt-based molecular sieve is 1.5% Co-BEA.

It can be seen that the catalytic activity on BEA is the best, which is the same as that on Fe-based molecular sieves. For further exploration, the catalytic oxidation activity of tert-butanol with 0.25~2% iron-based and cobalt-based BEA is investigated in the following section.

#### 3.2.3. Activity Comparison of Fe-BEA with Different Concentrations

**Figure 8:**
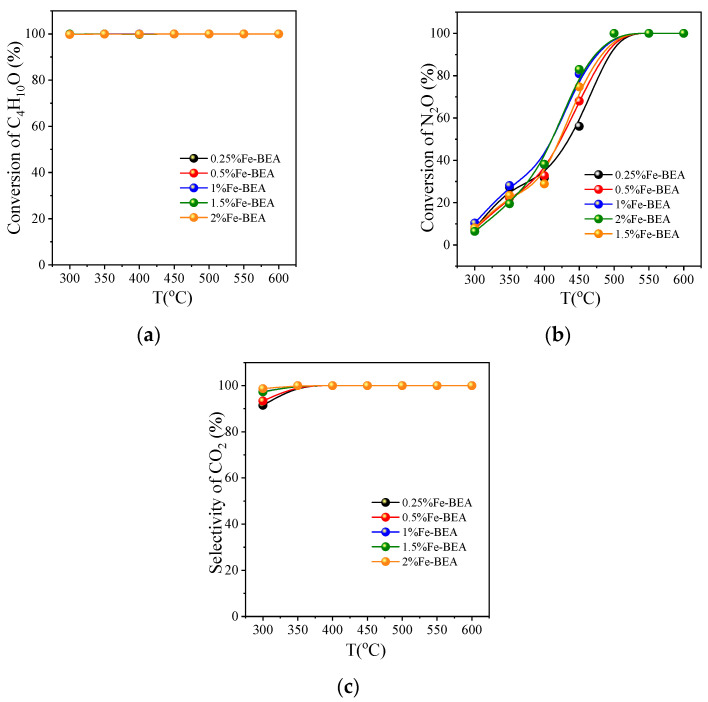
Catalytic oxidation of C_4_H_10_O on Fe-BEA with different concentrations: (**a**) the conversion of C_4_H_10_O; (**b**) the conversion of N_2_O; (**c**) the selectivity of CO_2_; reaction conditions: GHSV = 8000 h^−1^, C_4_H_10_O:N_2_O:O_2_:He = 0.2:7:5:87.8.

Figure 8a–c depict the reaction of the catalytic oxidation of C_4_H_10_O on Fe-BEA with different concentrations as a function of temperature. The research objects are BEA molecular sieves with 0.25~2% Fe-loaded concentration (0.25% Fe-BEA, 0.5% Fe-BEA, 1% Fe-BEA, 1.5% Fe-BEA, and R-2% Fe-BEA). When the reactant concentrations are C_4_H_10_O 2000 ppm, N_2_O 70,000 ppm, and O_2_ 50,000 ppm, it can be concluded that the conversion rates of C_4_H_10_O on the molecular sieve of 0.25~2% Fe-BEA are all above 99.9%, seen in Figure 8a. As for the conversion of N_2_O, the order of catalytic activity is different in the low-temperature section and the middle- and high-temperature sections. The catalytic performance of 1% Fe-BEA is slightly better at 300~350 °C, while the other conditions are the same, except that the concentration of supported iron is different. At 400~600 °C, the best conversion effect of N_2_O on 1.5% Fe-BEA is at 450 °C, and the conversion rate of N_2_O can reach 83.0%; at this time, the conversion rate of N_2_O on 1% Fe-BEA is 80.9%, and the conversion of N_2_O on Fe-BEA with different concentrations all reached 100% at 500 °C. In general, the N_2_O conversion rate of 1.5% Fe-BEA is higher than that of the other zeolites. In Figure 8c, it is shown that the selectivity of CO_2_ is very high, all above 90%. This shows that the oxidation of BEA is sufficient, so that most of the tert-butanol is converted into carbon dioxide. Overall, 1.5% Fe-BEA has the best performance.

#### 3.2.4. Activity Comparison of Co-BEA with Different Concentrations

**Figure 9:**
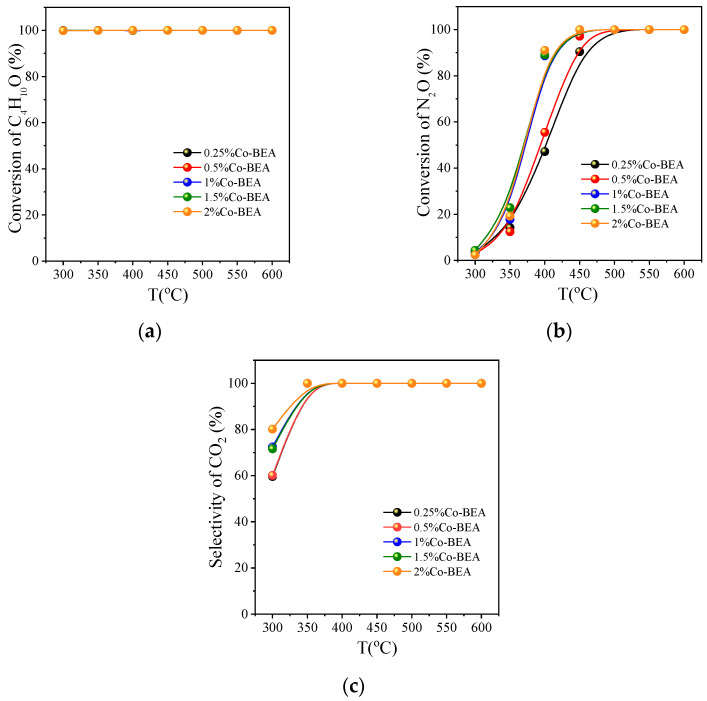
Catalytic oxidation of C_4_H_10_O on Co-BEA with different concentrations: (**a**) the conversion of C_4_H_10_O; (**b**) the conversion of N_2_O; (**c**) the selectivity of CO_2_; reaction conditions: GHSV = 8000 h^−1^, C_4_H_10_O:N_2_O:O_2_:He = 0.2:7:5:87.8.

Figure 9a–c depict the variation in the oxidation results of tert-butanol on different Co-BEA with temperature. The five concentrations of Co-BEA in Figure 9a were almost completely converted to tert-butanol. N_2_O begins to decompose at 300 °C and then oxidizes tert-butanol. However, the decomposition amount of N_2_O is less at a low temperature. Therefore, as an oxidant, O_2_ oxidizes tert-butanol in the low-temperature section. This is followed by N_2_O oxidation. The active oxygen in N_2_O can combine with cobalt in the catalyst to form α-Co. This enables the final oxidation of tert-butanol to CO_2_. The best catalytic activity in Figure 9b is 2% Co-BEA, followed by 1.5% Co-BEA. At 400 °C, the conversion rate of N_2_O at 2% Co-BEA is 91.1% and reaches 100% at 450 °C. At 300 °C, it can be seen in Figure 9c that the selectivity of CO_2_ decreases. Due to the inadequate oxidation of tert-butanol at a low temperature, a part of tert-butanol is oxidized to CO, resulting in the decreased selectivity of CO_2_. The selectivity of CO_2_ reaches 100% at 450 °C. Combined with the characterization results, the Co^2+^ content of 2% Co-BEA is the highest. This indicates that 2% Co-BEA has the most active sites, and Co^2+^ binds to α-O more easily, thus completely oxidizing tert-butanol to CO_2_. Therefore, 2% Co-BEA has the best catalytic effect.

## 4. Conclusions

The collaborative purification of N_2_O and tert-butanol was carried out over a series of zeolite catalysts, including FER, MOR, ZSM-5, Y, and BEA, wherein the BEA exhibited the best activity after being loaded with 1.5% Fe and 2% Co. The N_2_O conversion rate of 1.5% Fe-BEA can reach 83.0% at 450 °C and reach 100% at 500 °C, the conversion of tert-butanol was 100%, and the selectivity of CO_2_ was 100% at 350 °C. Combined with the results of characterization and activity evaluation experiments, 1.5% Fe-BEA had the highest Fe^3+^ content and the most active sites, and catalytic reactions are more likely to occur here. Therefore, 1.5% Fe-BEA had the best catalytic performance. It is a similar scenario for Co-BEA. More Co^2+^ and better catalytic activity was seen for 2% Co-BEA. The conversion rate of N_2_O on 2% Co-BEA was 91.1% at 400 °C and reached 100% at 450 °C, the conversion of tert-butanol reached 100% at 300 °C, and the selectivity of CO_2_ reached 100% at 450 °C. In this study, N_2_O and tert-butanol in the reactants were simultaneously treated harmlessly in the end treatment process of caprolactam exhaust emission. Research on the synergistic purification of N_2_O and tert-butanol is expected to be applied in the exhaust gas treatment device of industrial caprolactam.

## Figures and Tables

**Table 1:** Textural properties of H-BEA, Fe-BEA, and Co-BEA.

Samples	Surface Area m^2^/g	Micropore Volume cm^3^/g	Average Pore Diameter nm
H-BEA	562.0	0.18	15.0
0.25% Fe-BEA	490.1	0.14	14.0
0.5% Fe-BEA	463.7	0.15	15.8
1% Fe-BEA	449.8	0.13	14.7
1.5% Fe-BEA	414.7	0.11	13.2
2% Fe-BEA	421.1	0.15	15.2
0.25% Co-BEA	544.31	0.17	16.70
0.5% Co-BEA	561.71	0.17	14.85
1% Co-BEA	471.31	0.14	15.80
1.5% Co-BEA	488.65	0.14	15.79
2% Co-BEA	483.66	0.13	14.09

## Data Availability

Not applicable.

## Supplementary Materials

The following supporting information can be downloaded at https://www.mdpi.com/article/10.3390/ijerph20064902/s1: Figure S1: Effect of oxygen on N_2_O conversion in N_2_O and tert-butanol synergetic purification system over 1.5% Co-BEA; Figure S2: The selectivity of N_2_ on 1.5% Fe-BEA; Figure S3: Pulse test results of tert-butanol products oxidized by N_2_O at 300 °C on 1.5% Fe-BEA; Figure S4: Long time (30 h) reaction tests for 1.5% Fe-BEA and 1.5% Co-BEA samples at 600 °C: (a) the conversion of C_4_H_10_O on 1.5% Fe-BEA; (b) the conversion of N_2_O on 1.5% Fe-BEA; (c) the conversion of C_4_H_10_O on 1.5% Co-BEA; (d) the conversion of N_2_O on 1.5% Co-BEA; Figure S5: DFT simulation results of N_2_O oxidation of tert-butanol on Fe-BEA; Figure S6: The structure model of N_2_O oxidation of tert-butanol on Fe-BEA; Table S1: Reaction path of N_2_O oxidation of tert-butanol on Fe-BEA.
