# Supplementary material for: Collaborative Purification of Tert-Butanol and N2O over Fe/Co-Zeolite Catalysts
Source: Int J Environ Res Public Health. 2023 Mar 10;20(6):4902. doi: 10.3390/ijerph20064902 (PMC10049020; doi:10.3390/ijerph20064902)
Supplement: Supplementary file 1 [file ijerph-20-04902-s001.zip › ijerph-2236222-supplementary.pdf]

Supporting information

Collaborative Purification Tert-butanol and N_2O over Fe/Co-Zeolite Catalysts

Ruiqi Wu, Ning Liu, Chengna Dai, Ruinian Xu, Ning Wang, Gangqiang Yu, Biaohua Chen*

Faculty of Environment and Life, Beijing University of Technology, Beijing, 100124, China

* Correspondence: liuning@bjut.edu.cn

Table of Content

Figure S1. Effect of oxygen on N_2O conversion in N_2O and tert-butanol synergetic purification system over 1.5% Co-BEA. **S1**

Figure S2. The selectivity of N_2 on 1.5%Fe-BEA. **S1**

Figure S3. Pulse test results of tert-butanol products oxidized by N_2O at 300°C on 1.5% Fe-BEA. **S1**

Figure S4. Long time (30 h) reaction tests for 1.5%Fe-BEA and 1.5%Co-BEA samples at 600°C: (a) the conversion of $C_4H_{10}O$ on 1.5%Fe-BEA; (b) the conversion of N_2O on 1.5%Fe-BEA; (c) the conversion of $C_4H_{10}O$ on 1.5%Co-BEA; (d) the conversion of N_2O on 1.5%Co-BEA. **S2**

Figure S5. DFT simulation results of N_2O oxidation of tert-butanol on Fe-BEA. **S2**

Figure S6. The structure model of N_2O oxidation of tert-butanol on Fe-BEA. **S3**

Table S1. Reaction path of N_2O oxidation of tert-butanol on Fe-BEA. **S3**

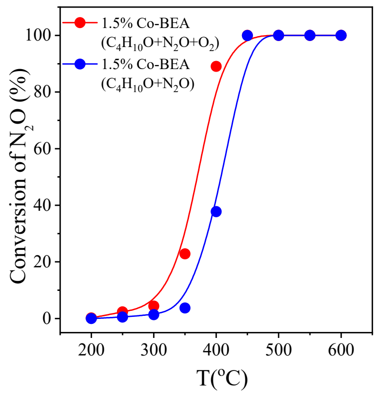

Figure S1. *Effect of oxygen on N2O conversion in N2O and tert-butanol synergetic purification system over 1.5% Co-BEA.*

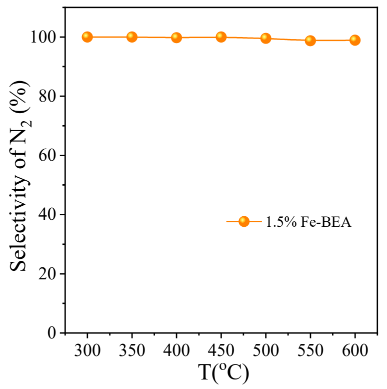

Figure S2. *The selectivity of N2 on 1.5%Fe-BEA.*

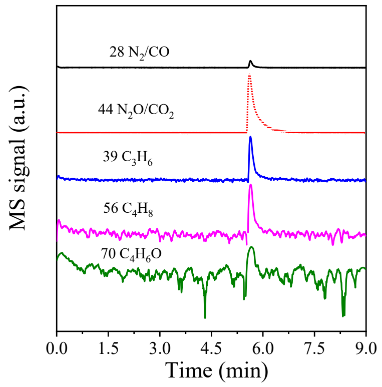

Figure S3. *Pulse test results of tert-butanol products oxidized by N2O at 300°C on 1.5% Fe-BEA.*

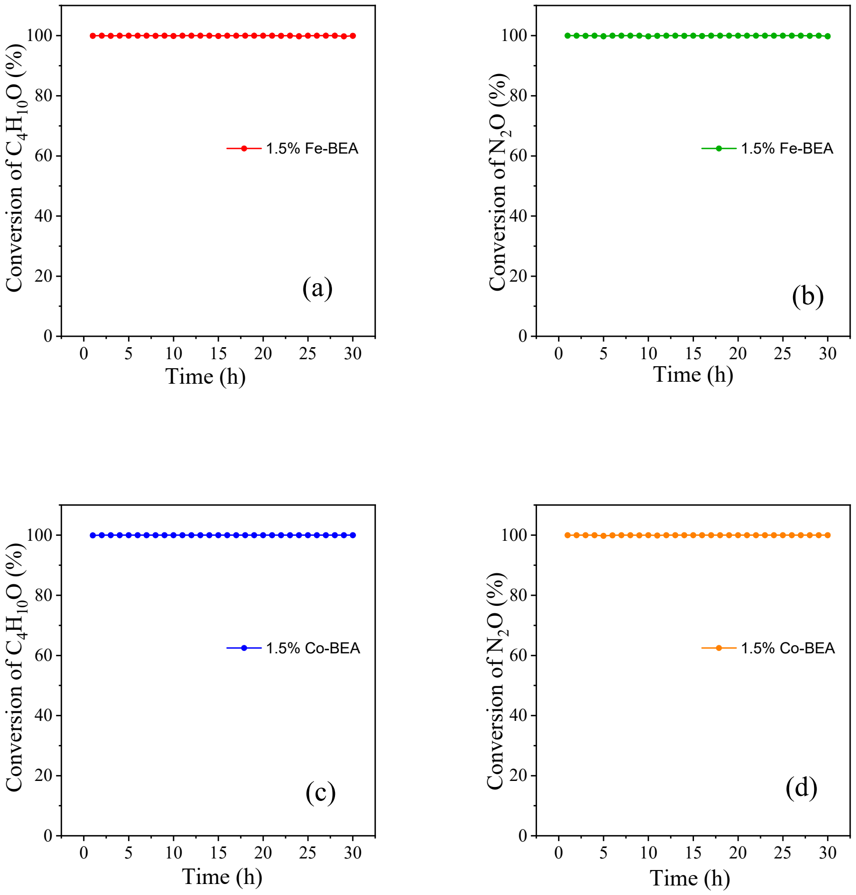

Figure S4. *Long time (30 h) reaction tests for 1.5%Fe-BEA and 1.5%Co-BEA samples at 600°C: (a) the conversion of $C_4H_{10}O$ on 1.5%Fe-BEA; (b) the conversion of N_2O on 1.5%Fe-BEA; (c) the conversion of $C_4H_{10}O$ on 1.5%Co-BEA; (d) the conversion of N_2O on 1.5%Co-BEA.*

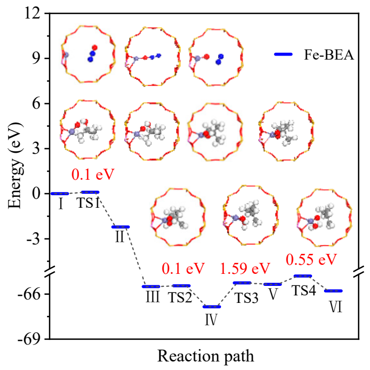

Figure S5. *DFT simulation results of N_2O oxidation of tert-butanol on Fe-BEA.*

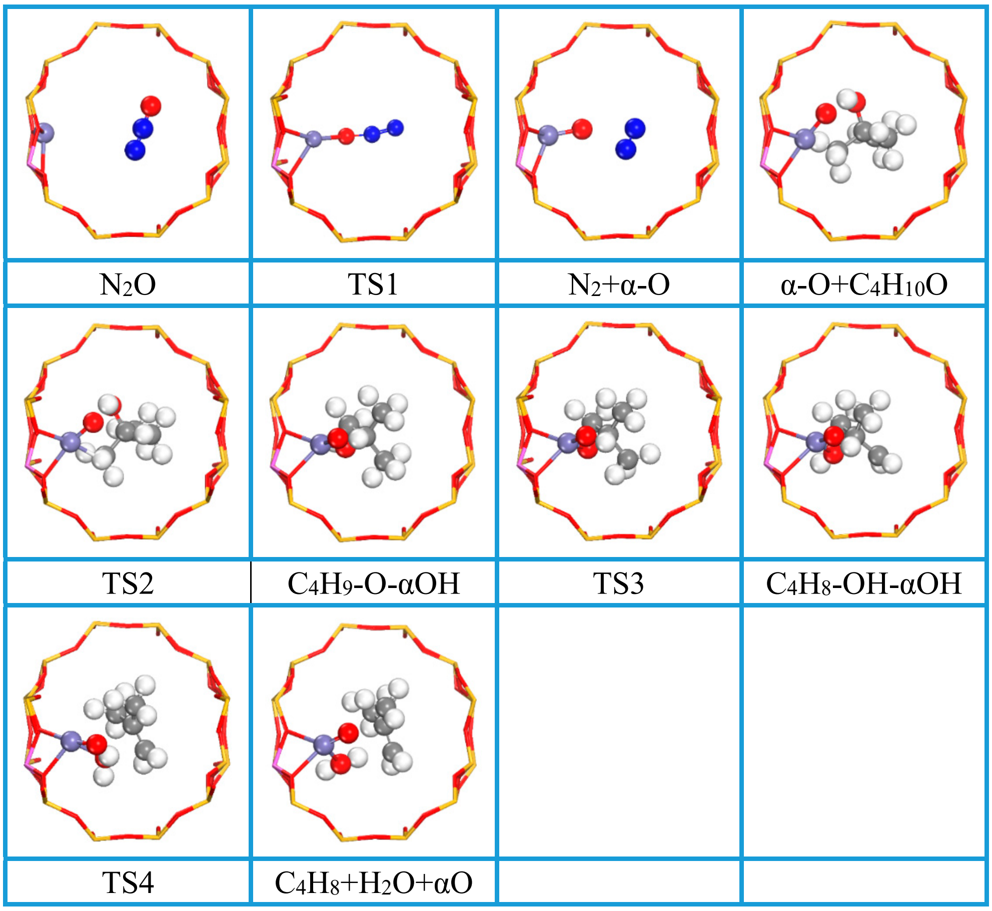

Figure S6. *The structure model of N_2O oxidation of tert-butanol on Fe-BEA.*

Table S1. *Reaction path of N_2O oxidation of tert-butanol on Fe-BEA.*

	Reaction path
I →TS1 → II	N_2O → N_2+α-O
III →TS2 → IV	α-O+$C_4H_{10}O$ → C_4H_9-O-αOH
IV →TS3 → V	C_4H_9-O-αOH → C_4H_8-OH-αOH
V →TS4 → VI	C_4H_8-OH-αOH → C_4H_8+H_2O+αO
